# The Effects of Thoracic Epidural Analgesia during Percutaneous Radiofrequency Ablation for Hepatocellular Carcinoma

**DOI:** 10.1155/2018/4354912

**Published:** 2018-11-19

**Authors:** Eun-Ji Choi, Yun-Mi Choi, Hye-Jin Kim, Hwoe-Gyeong Ok, Eun-Jung Chang, Hee-Young Kim, Ji-Uk Yoon, Kyung-Hoon Kim, Gyeong-Jo Byeon

**Affiliations:** ^1^Department of Anesthesia and Pain Medicine, Pusan National University Yangsan Hospital, Pusan National University School of Medicine, Busan, Republic of Korea; ^2^Research Institute for Convergence of Biomedical Science and Technology, Pusan National University Yangsan Hospital, Busan, Republic of Korea

## Abstract

**Background:**

Percutaneous radiofrequency ablation (PRFA) is a useful and safe treatment for hepatocellular carcinoma (HCC). Pain management, during and after PRFA, is a critical component of patient care.

**Objectives:**

This study reviewed the efficacy of thoracic epidural analgesia, during and after PRFA, for patients with HCC.

**Study Design:**

A retrospective, observational chart review.

**Setting:**

Tertiary medical center/teaching hospital.

**Methods:**

Patients who had undergone PRFA for HCC in the past 5 years were divided into two groups, based on the type of anesthesia administered: thoracic epidural anesthesia group (Group E) and local anesthesia with monitored anesthesia care group (Group C). We retrospectively reviewed changes in the numeric rating scale (NRS) score during and after PRFA, opioid consumption, length of the procedure, length of hospital stay, changes in blood pressure during PRFA, and the incidence of adverse events.

**Results:**

The NRS score in Group E was significantly lower than that in Group C (*P* < 0.05). The opioid consumption in Group E was lower than that in Group C after PRFA (*P* < 0.05). The procedure time was shorter in Group E (*P* < 0.05). Neither of the groups showed significant difference with respect to the length of hospital stay and the incidence of respiratory depression, fever, and blood pressure elevation. The incidence of nausea, vomiting, and voiding difficulty was higher in Group E.

**Limitations:**

This study is limited by its retrospective design.

**Conclusions:**

Thoracic epidural analgesia was associated with shorter procedure times, lower postprocedural pain, and lower opioid consumption during and after PRFA for HCC.

## 1. Introduction

Hepatocellular carcinoma (HCC) is a common type of cancer worldwide, with a poor prognosis [1, 2]. Although percutaneous radiofrequency ablation (PRFA) is a useful and safe method that is used extensively for treating hepatocellular carcinoma (HCC), it requires the use of anesthesia and analgesics, as the interventional radiologist typically requires the patients to cooperate in order to determine the tumor location and precise PRFA performance [3, 4].

Hepatic PRFA has been performed under various conditions, such as general anesthesia, intravenous anesthesia, epidural anesthesia, and thoracic paravertebral block [5–7]; however, it is usually performed under local anesthesia with intravenous sedation [8]. Nonetheless, many patients experience pain during and after the procedure. Although local anesthesia with intravenous sedation is the most commonly used and does not have special contraindications, intraoperative pain is poorly controlled using this method of anesthesia in rare cases and this can cause the practitioner to terminate the procedure. Moreover, due to the risk of developing hypertension from pain and, in some cases, respiratory depression, hypotension, and bradycardia from the anesthetics and analgesics used, patients should be closely monitored during the procedure, and the staff must be prepared for emergency situations [3, 6, 9]. Therefore, adequate pain relief during the procedure is most important. Inhibiting painful stress by thoracic epidural anesthesia might allow interventional radiologists to perform precise PRFA.

We performed a retrospective analysis on two groups of patients: the first group received thoracic epidural anesthesia during PRFA for HCC and the second group underwent the procedure under conventional local anesthesia with intravenous sedation. The groups were compared in an effort to identify a safer and more effective method of anesthesia.

## 2. Methods

### 2.1. Study Design

After institutional review board (05-2017-051) approval, a retrospective study was conducted utilizing electronic medical records to examine patients who underwent PRFA for HCC between January 2012 and December 2016. The requirement for written informed consent was waived by the institutional review board. The trial is registered with Clinical Research Information Service KCT0002606.

Two hundred thirty-three patients were selected from the charts reviewed. The diagnosis of HCC used clinical practice guideline by the European Association for the Study of the Liver and European Organization for Research and Treatment of Cancer (EASL–EORTC) [10]. It was based on the noninvasive criteria or pathology: noninvasive criteria can only be applied to cirrhotic patients and are based on imaging techniques obtained by 4-phase multidetector CT scan or dynamic contrast-enhanced MRI. Diagnosis should be based on the typical hallmark of HCC (hypervascular in the arterial phase with washout in the portal venous or delayed phases). Pathological diagnosis of HCC is based on the recommendations of the International Consensus Group for Hepatocellular Neoplasia [11]. Immunostaining for glypican 3 (GPC3), heat-shock protein 70 (HSP70), and glutamine synthetase and/or gene expression (GPC3, lymphatic vessel endothelial hyaluronan receptor 1 [LYVE1], and survivin) are recommended to differentiate high-grade dysplastic nodules from early HCC. The criteria of patient selection in this study were the following: (1) early HCC, not suitable for surgery, and (2) less than 5 cm sized due to a significantly better control of the disease.

Patients were informed of the advantages and disadvantages of intravenous sedation and thoracic epidural anesthesia by an anesthetist before signing the consent form for anesthesia. Patients were also informed that though there is no particular contraindication for intravenous sedation, pain control may be difficult, and while thoracic epidural anesthesia may provide good pain control, it may be accompanied by rare side effects related to epidural anesthesia, such as back pain, urinary retention, and neurological complications such as spinal infarction. Patients were asked to choose one of the methods of anesthesia, except when epidural block was not contraindicated (e.g., during anticoagulant therapy or coagulopathy). Whether epidural block will be contraindicated or not was decided after a discussion among hepatologist, radiologist, and anesthesiologist.

The patients were divided into two groups. Group E (*n*=51) consisted of patients who underwent PRFA under thoracic epidural anesthesia. Group C (*n*=182) included patients who underwent PRFA under local anesthesia with intravenous sedation. Neither group received premedication. After preparing all artificial respiration equipment and drugs, 20-gauge peripheral intravenous cannulas were inserted into patients for drug and fluid administration. Patients were monitored using electrocardiography, pulse oximetry, and noninvasive blood pressure monitoring. In Group E, fluoroscopic guided 20-gauge epidural catheter (Perifix®; B. Braun Medical Inc., Allentown, PA, USA) was inserted at the thoracic level (T8-9 or T9-10); and fentanyl 1 *µ*g/kg and 0.2% ropivacaine 6–8 mL, were injected via the catheter, approximately 30 minutes before PRFA. In Group C, intravenous sedation was rendered with 1–2 *µ*g/kg fentanyl and 1 *µ*g/kg/h dexmedetomidine for 10 minutes, and maintained with continuous intravenous (IV) infusion of dexmedetomidine at the rate of 0.5–1 *µ*g/kg/h. In both groups, 10 mL of 1% lidocaine was injected at the procedural site. All patients were allowed to breathe spontaneously during PRFA. During the procedure, patients received supplemental oxygen (3 L/min) via a nasal cannula.

During the course of the procedure, intravenous fentanyl (1–2 *µ*g/kg) was given to both groups, whenever patients needed procedure-induced pain relief. When the 11-point numerical rating scale (NRS-11) score was higher than 4 and the patient requested analgesics after the procedure, additional pethidine 25 mg was injected. The number of patients requiring opioid analgesics was calculated by including the patients who received additional opioid (pethidine), during and within 24 h postprocedure.

### 2.2. Data Collection

To increase the accuracy of data collection, two different investigators reviewed patient charts and collected data. A third investigator analyzed the collected data. Data regarding opioid consumption during and after the procedure, changes in pain (NRS-11; 0-no pain; 10-worst imaginable pain) during and after procedure, the procedure time, length of hospital stay, and changes in blood pressure during the procedure were collected retrospectively. Hypertension was defined as blood pressure ≥140/90 mmHg even after administration of opioids. Preprocedure and first day postprocedure laboratory findings were recorded, and the model for end-stage liver disease (MELD) scores and Child–Pugh score were calculated. Data related to perioperative adverse events, such as respiratory depression (oxygen saturation ≤94% or respiratory rate <12 breaths/min), fever, nausea and vomiting, and voiding difficulty, were also collected.

### 2.3. Statistics

All statistical tests were two-sided, and the significance level for all parameters was 0.05. Statistical analysis was performed using PASW Statistics for Windows, version 18.0 (SPSS Inc., Chicago, IL, USA) and MedCalc®, version 9.0 (MedCalc Software, Oostende, Belgium). All continuous variable data were reported as mean ± standard deviation (SD). Patient numbers, underlying disease, tumor count and size, opioid consumption, and the incidence of adverse effects were presented in terms of numbers and proportions.

Data distribution was evaluated using the Kolmogorov-Smirnov test. Continuous variables were analyzed using the Student's *t*-test. Chi-square test or Fisher's exact test was used for analyzing underlying disease, tumor count and size, opioid consumption, and the incidence of adverse effects.

## 3. Results

**Figure 1:**
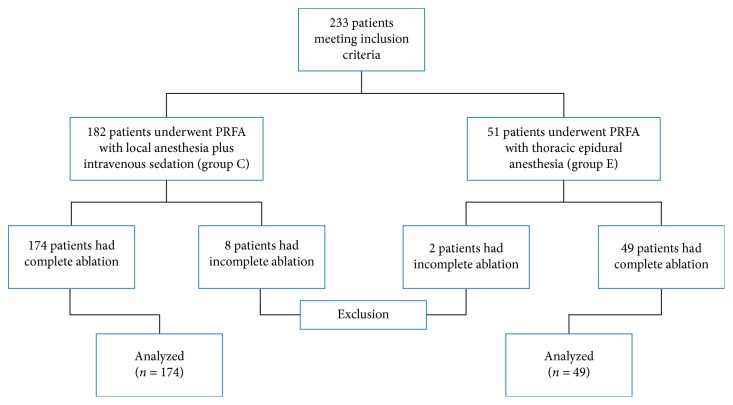
A study flow chart. PRFA = percutaneous radiofrequency ablation.

A total of 233 patients who underwent PRFA for HCC were enrolled in this retrospective study. They included 182 patients who had been given local anesthesia with intravenous sedation (Group C), and 51 patients who had been given thoracic epidural anesthesia (Group E). On chart review, 8 patients in Group C and 2 patients in Group E were excluded because of incomplete PRFA. Incomplete ablation was defined as the presence of arterial contrast enhancement and porto-venous washout within the PRFA site suggestive of residual tumor on CT imaging at 1 month after RFA. The incidence of incomplete PRFA was not statistically significant in either group. Incomplete PRFA showed in 8 out of 182 patients (4.4%) in Group C and 2 out of 51 patients (3.9%) in Group E. Thus, we analyzed 174 patients in Group C and 49 patients in Group E (Figure 1).

Demographic data collected from Groups C and E with regards to pre-, intra-, and postoperative factors showed no significant differences in gender, age, height, weight, underlying disease, tumor count and size, laboratory findings, Child–Pugh class, MELD score, and length of hospital stay between both groups. However, the procedure times in Groups C and E were 47.8 ± 18.0 and 41.4 ± 16.1 minutes, respectively; the length of the procedure was significantly shorter in Group E compared to that in Group C (*P* < 0.05, Table 1).

**Figure 2:**
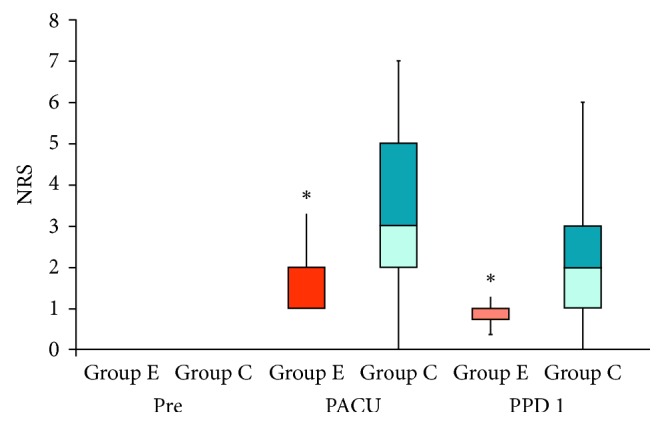
The 11-point numerical rating scale (NRS-11) score reported by patients after percutaneous radiofrequency ablation (PRFA). Pre = before procedure; PACU = postanesthesia care unit; PPD 1 = postprocedure day 1. ^*∗*^*P* < 0.05 compared with Group C.

Postprocedural pain scores in Groups C and E were 3.2 ± 2 and 1.8 ± 1, respectively, in the postanesthesia care unit (PACU), and 1.8 ± 1.5 and 1 ± 0.9, respectively, on day 1 postprocedure. The NRS score in the PACU and day 1 postprocedure in Group E was significantly lower than that in Group C (*P* < 0.05, Figure 2). The opioid consumption during PRFA was not different in both groups. Thirty out of 174 (17.4%) patients received intravenous fentanyl in Group C, and 6 out of 49 patients (12.2%) in Group E. However, during the 24-hour period following the procedure, 63 out of 174 patients (36.2%) in Group C requested additional pain medication, whereas only 2 out of 49 patients (4.1%) in Group E requested additional pain medication. So, Group E had significantly lower opioid consumption after PRFA compared to Group C (Table 2).

The incidence of adverse events such as respiratory depression, fever, and hypertension was not significantly different between the groups. Although not statistically significant, respiratory depression occurred in 10 out of 174 patients (5.7%) in Group C, while none of the patients in Group E developed respiratory depression. Hypertension also occurred in 7 out of 174 patients (4%) in Group C, while none of the patients in Group E developed hypertension. However, in Group E, the incidence of nausea and vomiting, and voiding difficulty were higher compared to those in Group C (*P* < 0.05, Table 3).

## 4. Discussion

We found that thoracic epidural anesthesia provided more effective pain relief than local anesthesia with intravenous sedation, in patients with HCC who underwent PRFA. Thoracic epidural analgesia resulted in lower postprocedural pain, lower need for opioids during and after PRFA, and shorter procedure times.

Treatment modalities for HCC include surgical resection, liver transplantation, and ablation [12]. Although hepatic resection is one of the primary treatments for HCC, surgical resection may not be feasible depending on the size, site, and number of tumors; vascular and extrahepatic lesions; and liver function [13–16]. Liver transplantation is challenging because of financial constraints, patient refusal to undergo surgery, risk of cardiopulmonary dysfunction, and lack of liver donors [11, 12]. PRFA is a treatment option for patients with HCC who are not candidates for transplantation, or who cannot undergo surgical resection [12, 17].

Hepatic PRFA is used extensively since it is less invasive, leading to fewer complications, and therefore, the procedure can be repeated when the lesion recurs [4, 15]. It can be performed under general anesthesia, total intravenous anesthesia, epidural anesthesia, or thoracic paravertebral block, but usually, it is performed under intravenous sedation [5–8]. However, pain caused by the procedure, even with sedation, is an issue. Pain can cause the practitioner to stop the procedure, or it could cause respiratory depression in the patient due to increased use of opioids. Patients may also suffer from postprocedure pain [3, 6].

In this retrospective study on pain during PRFA for HCC, we compared patients who received local anesthesia with intravenous sedation, with those who received thoracic epidural anesthesia. Patients from the thoracic epidural group showed significant decrease in postprocedural pain, and opioid consumption over a 24-hour period after the procedure. However, there was no difference between the two groups with respect to opioid consumption during the procedure. This could be attributed to the use of 0.2% ropivacaine during the procedure. Generally, ≥0.5% ropivacaine is used for intraoperative anesthesia [18]. Using a higher concentration could have resulted in a different outcome. Our findings indicated that thoracic epidural anesthesia is effective for postprocedural pain management.

With regard to the decrease in procedure time in Group E, we can infer that the procedure was performed more easily because intraoperative pain was well controlled, despite the fact that opioid consumption between the two groups was not significantly different. Although there was a difference in the procedure time, the opioids consumption was a measure of the number of additional patients due to postprocedural pain, except for the number of routinely administered in Group C. It is considered that these results were obtained by not administering opioids more than once due to fear of adverse effects such as respiratory depression, bradycardia, and so on.

The incidence of respiratory depression and hypertension was not statistically significant in either group. Respiratory depression occurred in 10 out of 174 patients (5.7%) in Group C, but not in patients in Group E. In addition, hypertension occurred in 7 out of 174 patients (4%) in Group C, but not in patients in Group E. These findings were noteworthy, and the possibility that statistically significant results could have been obtained with a higher number of patients in both groups cannot be dismissed.

Dexmedetomidine is an alpha-2 agonist, which is commonly used to sedate patients without tracheal intubation [19]. It does not cause respiratory depression, but can sometimes cause hypotension, bradycardia, and serious complications in rare cases [19, 20]. In this study, patients in Group E were not sedated, and, hence, those risks were reduced. In addition, there was no statistically significant difference in the incidence of hypertension between the two groups, which may be attributed to the hypotensive effect of dexmedetomidine, leading to reduction in the incidence of hypertension due to the analgesic effect of epidural anesthesia.

There are several reports on anesthetic methods during hepatic PRFA. One study performed right thoracic paravertebral block (TPVB) during PRFA on 20 patients and achieved postoperative analgesic effect [21]. However, this method can cause pain in the unblocked contralateral side. Another study compared monitored anesthetic care (MAC) and epidural anesthesia during PRFA, and reported that PRFA performed under MAC reduced the recurrence of HCC, but had no impact on survival rate [7]. However, their study was not focused on postprocedural pain. It should be kept in mind that our study focuses on postprocedural pain.

There was no statistically significant difference between the two groups with respect to the length of hospital stay. This was because postoperative pain was manageable with additional pain medication and was not severe enough to prolong the length of hospital stay. There was no statistically significant difference between the two groups with respect to fever as well. Moreover, the study did not find any differences in inflammatory responses as a result of the anesthetic methods used, in both groups.

The incidence of postprocedural nausea, vomiting, and voiding difficulty, was higher in Group E. We believe that these results represent the adverse effects of epidural anesthesia; however, no other serious complications were observed. Another disadvantage of epidural anesthesia is that it is contraindicated in cases involving infection or coagulation disorder, and therefore, it cannot be used in all patients [5]. Despite these disadvantages, thoracic epidural anesthesia reduced the procedure time and was effective against postprocedural pain. In this study, a high number of patients underwent local anesthesia with intravenous sedation instead of thoracic epidural anesthesia. This was because the patients selected local anesthesia with intravenous sedation when filling out the anesthesia consent form. Considering the benefits of thoracic epidural anesthesia identified in this study, it is necessary to fully explain the benefits of this method to doctors from other departments and patients and advise them to choose thoracic epidural anesthesia as the method of anesthesia.

Our study had some limitations. First, this study was a single-center retrospective study, and multicenter prospective studies are needed. Second, there were no data on surgeon satisfaction resulting from better patient cooperation due to pain reduction during the procedure. Therefore, surgeon satisfaction surveys are needed. It is believed that the percentage of incomplete ablation cases would decrease as the procedure becomes easier, which may also explain the difference in procedure time between the two groups. Lastly, the study was concluded 24 hours after the procedure, but it would be necessary to compare the complete ablation rates, recurrence, and survival rates between the two groups over a longer term.

In conclusion, thoracic epidural analgesia led to shorter procedure times, lower postprocedural pain, and lower opioid consumption during and after PRFA for HCC.

## Figures and Tables

**Table 1:** Patient characteristics.

Characteristics	Group C (*n*=174)	Group E (*n*=49)	*P* value
Sex (M/F)	128/46	37/12	
Age (years)	62.8 ± 9.8	65.4 ± 10.3	0.111
Height (cm)	163.5 ± 8.6	162.9 ± 7.7	0.666
Weight (kg)	64.1 ± 12.7	65.3 ± 12.9	0.587
*Underlying disease*			
Hypertension (%)	45 (25.9)	16 (32.7)	0.367
Diabetes mellitus (%)	49 (28.2)	8 (16.3)	0.099
Cardiovascular disease (%)	5 (2.9)	3 (6.1)	0.280
Respiratory disease (%)	2 (1.1)	1 (2.0)	0.632
Chronic kidney disease (%)	7 (4.0)	2 (4.1)	0.985
*Tumor count*			0.229
Less than 1 (1≤)	142 (81.6)	36 (73.5)	
2 or more (≥2)	32 (18.4)	13 (26.5)	
*Tumor size*			0.278
<2 cm	138 (79.3)	43 (87.8)	
2-3 cm	34 (19.5)	6 (12.2)	
3–5 cm	2 (1.1)	0 (0.0)	
Procedure time (min)	47.8 ± 18.0	41.4 ± 16.1^*∗*^	0.025
AST (before RFA) (IU/L)	51.4 ± 38.8	43.1 ± 27.5	0.161
ALT (before RFA) (IU/L)	39.5 ± 33.6	39.7 ± 41.2	0.972
AST (after RFA) (IU/L)	50.7 ± 34.8	43.8 ± 30.6	0.211
ALT (after RFA) (IU/L)	39.2 ± 29.7	36.4 ± 29.6	0.566
Child–Pugh class (A/B)	161/13	49/0	
MELD score	5.9 ± 3.9	4.6 ± 3.2	0.127
Length of hospital stay (day)	1.3 ± 0.9	1.4 ± 1.3	0.387

All measured values are presented as mean ± standard deviation or number of patients (%). AST = aspartate aminotransferase; ALT = alanine aminotransferase; MELD = Model for End-Stage Liver Disease. ^*∗*^*P* < 0.05 compared with Group C.

**Table 2:** Opioid consumption, during and after percutaneous radiofrequency ablation (PRFA).

	Group C (*n*=174)	Group E (*n*=49)	*P* value
Patients who received opioid during PRFA (%)	30 (17.2)	6 (12.2)	0.535
Patients who received opioid after PRFA (%)	63 (36.2)	2 (4.1)^*∗*^	<0.001

Values are the number of patients (%). ^*∗*^*P* < 0.05 compared with Group C.

**Table 3:** Incidence of adverse effects.

Characteristic	Group C (*n*=174)	Group E (*n*=49)	*P* value
Respiratory depression (%)	10 (5.7)	0 (0.0)	0.123
Nausea and vomiting (%)	29 (16.7)	16 (32.7)^*∗*^	0.024
Fever (%)	6 (3.4)	2 (4.1)	0.689
Voiding difficulty (%)	1 (0.6)	18 (36.7)^*∗*^	<0.001
Hypertension (%)	7 (4.0)	0 (0.0)	0.352

Values are the number of patients (%). ^*∗*^*P* < 0.05 compared with Group C.

## Data Availability

The data used to support the findings of this study are available from the corresponding author upon request.
